# Phylogenomic Analysis of Global Isolates of Canid Alphaherpesvirus 1

**DOI:** 10.3390/v12121421

**Published:** 2020-12-10

**Authors:** Andrew C. Lewin, Lyndon M. Coghill, Melanie Mironovich, Chin-Chi Liu, Renee T. Carter, Eric C. Ledbetter

**Affiliations:** 1 Veterinary Clinical Sciences, School of Veterinary Medicine, Louisiana State University, Baton Rouge, LA 70803, USA; mironovich1@lsu.edu (M.M.); cliu@lsu.edu (C.-C.L.); reneecarter@lsu.edu (R.T.C.); 2 Center for Computation and Technology, Louisiana State University, Baton Rouge, LA 70808, USA; lcoghill@cct.lsu.edu; 3 Department of Clinical Sciences, College of Veterinary Medicine, Cornell University, Ithaca, NY 14853, USA; ecl32@cornell.edu

**Keywords:** CHV-1, canine herpesvirus, canid alphaherpesvirus, phylogenetics, genomics, surveillance

## Abstract

Canid alphaherpesvirus 1 (CHV-1) is a widespread pathogen of dogs with multiple associated clinical signs. There has been limited prior investigation into the genomics and phylogeny of this virus using whole viral genome analysis. Fifteen CHV-1 isolates were collected from animals with ocular disease based in the USA. Viral DNA was extracted for Illumina MiSeq full genome sequencing from each isolate. These data were combined with genomes of previously sequenced CHV-1 isolates obtained from hosts in the UK, Australia and Brazil. Genomic, recombinational and phylogenetic analysis were performed using multiple programs. Two isolates were separated into a clade apart from the remaining isolates and accounted for the majority of genomic distance (0.09%): one was obtained in 2019 from a USA-based host (ELAL-1) and the other in 2012 from a host in Brazil (BTU-1). ELAL-1 was found to contain variants previously reported in BTU-1 but also novel variants in the *V57* gene region. Multiple non-synonymous variants were found in USA-based isolates in regions associated with antiviral resistance. Evidence of recombination was detected between ELAL-1 and BTU-1. Collectively, this represents evidence of trans-boundary transmission of a novel form of CHV-1, which highlights the importance of surveillance for this pathogen in domestic dog populations.

## 1. Introduction

Disease caused by CHV-1 is widespread in canine populations [1,2,3] and affects both neonatal and adult animals. The virus is a significant cause of sudden death in neonates [4], ocular disease [5], respiratory disease [6,7] and reproductive disease [8,9]. Despite this, there has been minimal genomic assessment of the virus to date.

CHV-1 is a varicellovirus of the herpesviridae family, which establishes latency following an initial infection period [10,11]. The virus has a worldwide distribution [12,13,14] and a high seroprevalence among various canine populations [1,2,3]. Diagnosis of disease is currently based on viral isolation, PCR or serology. Although symptomatic treatment of certain forms of CHV-1 (ocular disease) has progressed in recent years [15], minimal options are available for effectively treating the disease in neonatal animals. Vaccines are available but do not offer complete protection against infection [16,17]. Together, this means that disease surveillance and prevention are essential for control of this virus.

The structure of CHV-1 is similar to other herpesviruses, with unique long (U_L_) and short (U_S_) sequences flanked by terminal (TR_S_/TR_L_) and internal inverted (IR_S_/IR_L_) repeats. The overall length is approximately 125 kbp with 76 open reading frames [18]. Several complete/near-complete CHV-1 isolates have been sequenced and analyzed in the past [18,19,20]. Phylogenetic analysis of complete CHV-1 genomes has been previously been performed using a very limited number of isolates [18,20]. While most CHV-1 genomes were previously found to be mostly homogenous (0.005% distance in UK isolates), one isolate (BTU-1) obtained from a Brazilian host canid in 2012, was noted to be 0.34% distant from the others, mostly due to variants in the CHV-1 *UL50* protein sequence region [20].

Our objective was to characterize the genomes of circulating CHV-1 isolates in the USA and to relate their sequence characteristics to global isolates. We hypothesized that CHV-1 isolates obtained in the USA would be genomically homogenous but would separate into clades based on geography, as is the case for the related varicellovirus, feline herpesvirus 1 (FHV-1) [21].

## 2. Materials and Methods

### 2.1. Cells and Viruses

Virus isolation was performed in a routine manner using diagnostic samples collected from naturally infected host canids (with informed owner consent). Sample collection was performed by vigorously swabbing the conjunctival fornices of host animals using a flocked swab, which was then immediately placed into universal viral transport media (BD, NJ) for transport. Tubes containing swabs and viral transport medium were vortexed. Approximately 1.5 cc of viral transport medium was inoculated onto cells and allowed to absorb for 1 h with periodic rocking. Isolations were performed with A-72 canine cells (ATCC, CRL-1542, VA), Madin-Darby Canine Kidney (MDCK) cells (ATCC, CRL-2935, VA), and a laboratory-developed canine skin cell line in minimum essential medium-E with 10% fetal bovine serum, 5% serum replacement solution, 2% penicillin-streptomycin solution, 1% amphotericin B solution and 1% gentamicin sulfate solution. Cultures were incubated at 37 °C (5% CO_2_), checked at 24-h intervals for cytopathic effect (CPE), subcultured every 5–7 days, and held for up to 21 days. Cell cultures with CPE typical of CHV-1 were stained with anti-CHV-1 polyclonal antiserum conjugated to fluorescein isothiocyanate (CHV-1 direct fluorescent antibody conjugate, CJ-F-CHV-10ML, VMRD Inc, Pullman, WA, USA) [22] to confirm viral identification. At this point, each flask was subjected to 3 freeze/thaw cycles before the contents were transferred to a 15 cc conical tube and centrifuged at 600× *g* for 5 min at 4 °C. The supernatant was then removed and aliquoted for storage at −80 °C prior to bulk processing for sequencing.

Using a 500 µL volume of previously stored supernatant, a T25 flask (Thermo Fisher, Waltham, MA, USA) containing a monolayer of MDCK cells was subsequently infected in a similar manner for each isolate using Dulbecco’s Modified Eagle Media (DMEM) (Thermo Fisher, Waltham, MA, USA) containing 2% fetal bovine serum (Thermo Fisher, Waltham, MA, USA) and 1% penicillin/streptomycin (Thermo Fisher, Waltham, MA, USA). Following observation of 100% CPE, 3 freeze/thaw cycles and centrifugation (600× *g* for 5 min at 4°C), 200µL of the resulting supernatant was used for viral DNA extraction. Viral DNA was prepared using a commercial kit according to the manufacturer’s instructions (Purelink Viral RNA/DNA Mini Kit, Invitrogen, Carlsbad, CA, USA).

### 2.2. Sequencing

Extracted DNA was submitted to the Louisiana State University School of Veterinary Medicine GeneLab. DNA purity and concentration were assessed using a NanoDrop One Microvolume Spectrophotometer (Thermo Scientific, Waltham, MA, USA) and then processed using the Nextera DNA Flex Library Prep Kit (Illumina Inc., San Diego, CA, USA) with modifications specific for the 100–500 ng DNA input range. Quality and quantity of the finished libraries were assessed using a Fragment Analyzer Instrument (Advanced Analytical) and dsDNA HS Assay Kit, respectively. Libraries were pooled, standardized to 10 μM and paired end sequencing was performed using the Illumina MiSeq Sequencer and a MiSeq 500 bp (v2) sequencing kit (MS-102-2003).

### 2.3. Genome Assembly

Reference-based assembly was performed using Geneious Prime ver 2020.2.4. Paired end reads were trimmed using BBDuk adapter/quality trimming ver 38.84 (right end, Kmer length = 27, maximum substitutions = 1, minimum quality = 20, minimum overlap = 20, minimum length = 20). Trimmed paired end reads were then assembled to the reference sequence for CHV-1 (0194, Genbank accession NC_030117). A consensus sequence was extracted from the aligned reads with gaps filled with “N’s”. Genomes were annotated and submitted to Genbank using Geneious Prime ver 2020.2.4.

### 2.4. Viral Genome Alignments

Alignments were created using MAFFT ver 7.450 [23] within Geneious Prime ver 2020.2.4. In all instances, the default parameters were used; a scoring matrix of 1 PAM/k = 2, gap penalty of 1.53 and offset value of 0.123. Alignments created included all whole CHV-1 genomes (USA + UK + Brazil + Australia), all whole CHV-1 genomes plus a Feline herpesvirus (FHV-1) outgroup (C-27, Genbank accession NC_013590.2) and the isolated CHV-1 *V57* gene region from 3 CHV-1 genomes (0194, ELAL-1 and BTU-1). An additional alignment using the same 3 complete CHV-1 genomes (0194, ELAL-1 and BTU-1) was also created.

### 2.5. Variant Analysis

Variant analysis was performed using the Geneious variant finder (Geneious Prime ver 2020.2.4) in regions with a minimum coverage = 100, minimum variant frequency (proportion of reads matching variant) = 0.25, maximum variant *p*-value = 10^−6^ and minimum strand-bias (disagreement between the forward and reverse strand) *p*-value =10^−5^ when exceeding 65% bias. Variants were called by comparing each sequenced isolate to the reference genome (CHV-1 0194). Linear regression with viral gene length as the repressor and the total number of the variants per gene as the outcome (with 99% confidence limits) was constructed using JMP Pro 15.0.0 (SAS Institute Inc., Cary, NC, USA).

Regions of genomic distance between 3 CHV-1 isolates (0194, ELAL-1 and BTU-1) were visualized using RDP ver 4.100 [24] using the manual distance plot function (window = 1200, step = 500, transition:transversion rate ratio = 2, coefficient of variation = 1, Jin and Nei model [25]).

### 2.6. Phylogenetic and Recombination Analysis

An alignment containing all whole CHV-1 genomes plus a Feline herpesvirus (FHV-1) outgroup was processed using ModelFinder [26] via IQ-Tree 2 ver 1.6.12 [27] to automatically select the best-fit model (TVM+F+G4). The resultant maximum likelihood tree was viewed using Splitstree ver 4.16.1 [28]. Recombination analysis was performed using RDP ver 4.100 [24] on an aligned set of CHV-1 genomes using a manual bootscan (window = 1200, step = 500, replicates = 100, 70% cutoff, Jin and Nei model [25]), RDP, GENECONV, MaxChi and Chimaera.

Pairwise genomic distances were calculated using MEGAX [29] with the gamma distribution model (5), partial deletion of gaps and 1000 bootstrap replicates.

### 2.7. Sequence Accession Numbers

All isolates used for analysis are shown in Table 1 and are available on the GenBank website (https://www.ncbi.nlm.nih.gov/genbank/).

## 3. Results

### 3.1. Sequencing and Genome Assembly

A total of 15 isolates of CHV-1 were sequenced using Illumina MiSeq. A further five previously sequenced CHV-1 genomes were included in the analysis (Table 1). The majority of isolates sequenced for this study were obtained from animals in the NY, USA region and all were taken from animals with ocular disease (conjunctivitis and keratitis). A variety of host breeds were represented, with most (7/15) being beagles. Almost all (6/7) of these beagles were housed together in New York State and had samples taken as part of an investigation into a CHV-1 disease outbreak. Both juvenile and adult host animals are represented for the 15 isolates sequenced for this study, with the youngest being approximately 8 weeks old (ELAL-13) and the oldest being 12 years old (ELAL-12).

The details of sequencing are shown in Table 2. The total number of reads ranged from 1,292,262 (ELAL-3) to 3,533,658 (ELAL-10). The number of reads mapped ranged from 155,016 (ELAL-1) to 856,992 (ELAL-14). The mean mapped read length ranged from 201.1 (ELAL-12) to 228.6 (ELAL-2). Mean coverage across the genomes ranged from 290× (ELAL-1) to 1509× (ELAL-6). Coverage was excellent throughout the length of the genomes, with rare regions of less than 100× coverage in several isolates (Figure S1). The average G-C content was 31.6%, and this value was fairly consistent along the length of all genomes (with one elevation 36 kb from the left end of the genome of approximately 74% G-C content). No relationship between G-C content and coverage was observed.

### 3.2. Variant Analysis

Variants were found to be evenly distributed throughout the length of the genomes and are summarized in Table 3 and Table S1. A total of 237 unique synonymous and 233 unique non-synonymous variants were found across the genomes. The most unique synonymous variants were located in the *RS1* gene region (22) and the most unique non-synonymous variants (42) were located in the *UL36* gene region. The number of unique synonymous variants within each isolate ranged from 4 (ELAL-2, ELAL-8) to 188 (ELAL-1). The number of unique non-synonymous variants ranged from 17 (ELAL-8) to 154 (ELAL-1). The total number of variants for each gene is plotted against gene length in a regression analysis in Figure S2. A single gene (*UL50*/53 variants/918 bp) was found to contain a higher-than-expected number of variants based on gene length.

Non-synonymous variants were detected in *UL23* (thymidine kinase), *UL30* (DNA polymerase catalytic subunit) and *UL42* (DNA polymerase processivity subunit), all regions which are targeted by commonly used antiviral medications [30]. All sequenced isolates apart from 4 (ELAL-6, ELAL-7, ELAL-12, ELAL-15) contained non-synonymous variants in *UL30*, whereas only two isolates contained non-synonymous variants in *UL42* (ELAL-1, ELAL-6). Only one isolate (ELAL-1) contained non-synonymous variants in *UL23*, *UL30* and *UL42*.

### 3.3. Phylogenetic and Recombination Analysis

**Figure 1:**
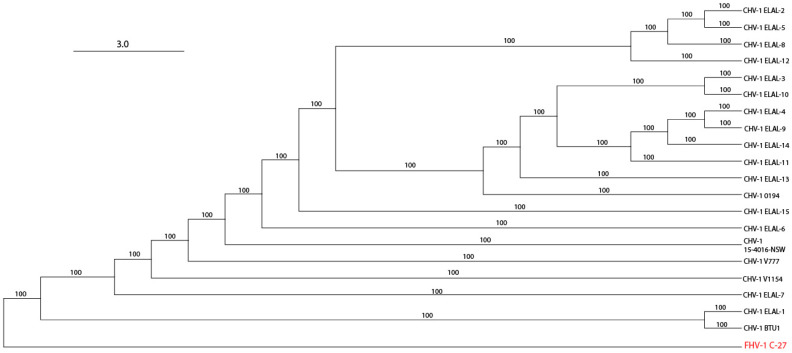
Maximum likelihood tree of all analyzed CHV-1 isolates with an FHV-1 C-27 outgroup (in red). Branch labels represent bootstrap support. Note the separation of two CHV-1 isolates (ELAL-1 and BTU-1) from the remaining CHV-1 isolates. CHV-1 ELAL-1 was obtained from a host animal in Texas in 2019 and BTU-1 was obtained from a host animal in Brazil in 2012.

The transversional model (TVM+F+G4) [31] was automatically chosen based on the Bayesian information criterion score (BIC) using ModelFinder [26] within IQ-Tree 2 ver 1.6.12 [27]. The resultant tree file was used to create a maximum likelihood tree (Figure 1). All bootstrap support values were 100, suggesting strong support for the recovered clade relationships. The maximum likelihood tree was visualized using Geneious Prime ver 2020.2.4.

**Figure 2:**
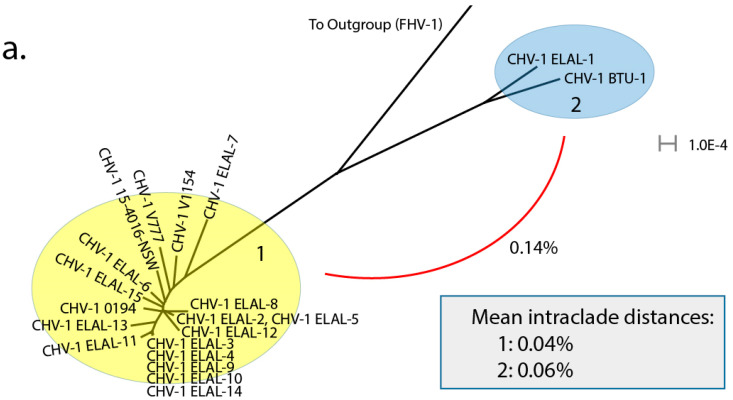

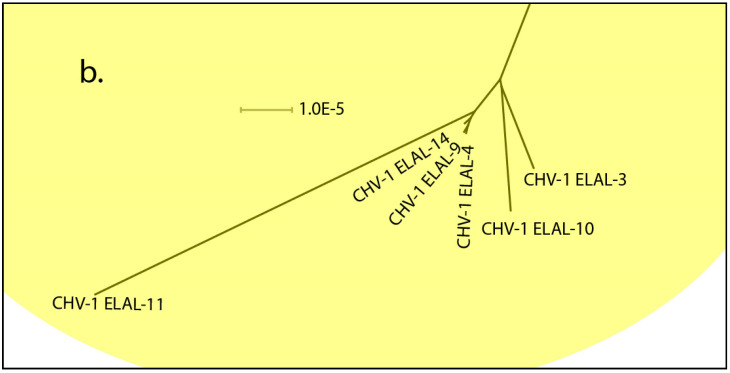
(**a**) Phylogenetic network of all analyzed CHV-1 isolates with a FHV-1 (C-27) outgroup. Two distinct clades are visible: 1 (yellow) and 2 (blue). The interclade distance (0.14%) is illustrated with a red line and was calculated using an alignment of consensus sequences (MAFFT ver. 7.450) from each clade. The mean intraclade distances were calculated using MEGAX. (**b**) Detail of subsection of clade 1 from Figure 2a. All isolates shown in this subsection were obtained from the same outbreak of CHV-1 in New York State in 2008. As expected, the genomes of these particular CHV-1 isolates are extremely homogenous.

The same tree file was also visualized using Splitstree ver 4.16.1 [28] (Figure 2) to further elucidate clade structure. Two clades were visualized. Clade 2 contained two isolates: ELAL-1 (a CHV-1 isolate collected from a host in 2019 in San Antonio, TX, USA) and BTU-1 (a CHV-1 isolate collected from a host in 2012 in Brazil). Clade 1 contained 18 isolates, most of which originated from host animals in the USA. Clade 1 also contained previously sequenced isolates from the UK [18] and Australia [19]. As expected, isolates obtained from multiple host animals in the same colony during an outbreak (ELAL-3, ELAL-4, ELAL-9, ELAL-10, ELAL-11, ELAL-14) were extremely homogenous and were grouped closely together (Figure 2b).

The overall mean genomic distance for all isolates sequenced for this study was 0.09% (calculated using MEGAX). Interclade genomic distance was 0.14%, as calculated using an alignment of consensus sequences (MAFFT ver. 7.450) from each clade. Clades were designated based on previously outlined criteria for varicelloviruses [20]. Intraclade distance was slightly lower in Clade 1 (0.04%) than in Clade 2 (0.06%).

**Figure 3:**
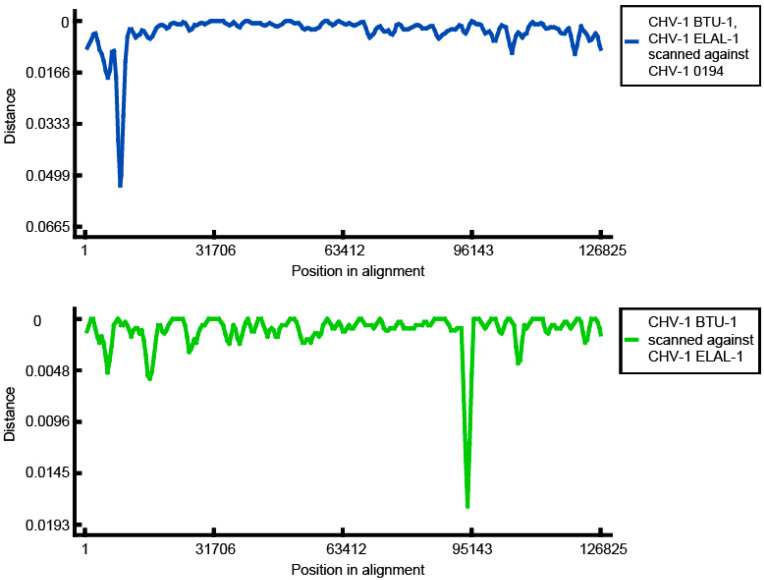
Genomic distance plotted against sequence position for CHV-1 isolates. An alignment containing 3 isolates (ELAL-1 (Clade 2), BTU-1 (Clade 2) and 0194 (Clade 1)) was processed in RDP4 ver 4.100. Results are for a manual distance plot with a window size of 1200, step size of 500 using the Jin and Nei model. The top graph (blue line) clearly illustrates a deep trough at approximately position 9kb in the alignment when either of the clade 2 isolates are scanned against the CHV-1 reference genome (0194, Clade 1). The bottom graph (green line) shows a deep trough at approximately position 93kb in the alignment when Clade A isolates are compared. This corresponds with the *V57* gene region.

To further investigate the origin of genomic variation between Clade 1 and Clade 2, a distance plot (Figure 3) was created using RDP ver 4.100 [24] with an alignment containing 3 CHV-1 genomes; ELAL-1 (Clade 2), BTU-1 (Clade 2) and 0194 (Clade 1). When either BTU-1 or ELAL-1 were scanned against 0194, a trough representing the increased genomic distance was visualized approximately 9 kb from the left end of the genome. This has been previously reported for BTU-1 and is due to variants in the *UL50* deoxyuridine triphosphatase gene region in this isolate [20]. When BTU-1 and ELAL-1 were scanned against each other, a deep trough 93 kb from the left end of the genome was visualized, which corresponds with the CHV-1 *V57* protein region.

**Figure 4:**
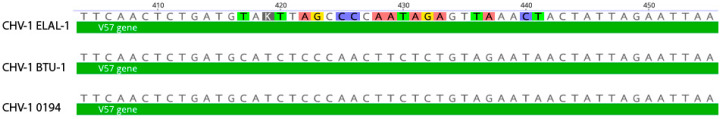
Variant in CHV-1 ELAL-1 isolate in the *V57* gene region. A clearly identifiable region of low identity was detected centered around position 430 in the *V57* gene which corresponded to a novel variant in CHV-1 ELAL-1.

An alignment containing the *V57* gene of the same three isolates (BTU-1, ELAL-1, 0194) was created and visualized using Geneious Prime ver 2020.2.4 (Figure 4). A novel variant between positions 417 and 441 in the *V57* gene of ELAL-1 was detected. Apart from this variant, the *V57* sequences of the three isolates were almost identical.

**Figure 5:**
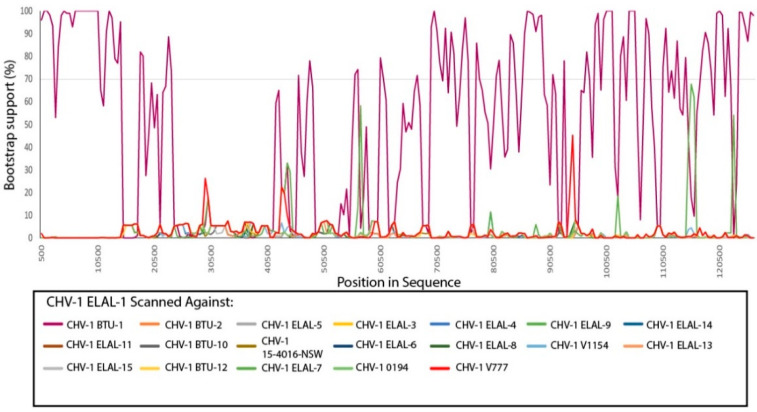
Manual bootscan results for recombination detection of CHV-1 ELAL-1 against all other analyzed isolates. A cutoff value of 70% bootstrap support is shown on the graph. Clear evidence of recombination was found between CHV-1 ELAL-1 and fellow Clade 2 isolate CHV-1 BTU-1, but not between CHV-1 ELAL-1 and any of the remaining isolates.

Recombination detection was performed on an alignment containing all available CHV-1 genomes in RDP ver 4.100 [24]. A PHI test demonstrated very good evidence of recombination in the alignment (*p* < 0.00001). Evidence for recombination between ELAL-1 and BTU-1 was abundant; RDP, GENECONV, Bootscan (Recscan), MaxChi and Chimaera supported this hypothesis. The results of a manual bootscan performed in RDP4 are shown in Figure 5. Manual bootscans were also performed for all other isolates versus the remaining isolates, with complete results shown in Table S2. Using this technique, multiple isolates did not demonstrate any evidence of recombination with other isolates, including all isolates shown in Figure 2b from the same outbreak in New York State in 2008 (ELAL-3, ELAL-4, ELAL-9, ELAL-10, ELAL-11, ELAL-14) as well as ELAL-6, ELAL-12, 0194 and 15-4016-NSW. The remaining Clade 1 isolates (ELAL-2, ELAL-5, ELAL-7, ELAL-8, ELAL-13 and ELAL-15, V1154, V777) all demonstrated some evidence of recombination with other Clade 1 isolates with bootstrap support of over 70%. There was no evidence of recombination between clades.

## 4. Discussion

To the authors’ knowledge, the present study represents the first known description of the genomics and phylogeny of CHV-1 isolates obtained from USA-based host canids. Overall, most of the isolate genomes studied were very similar to previously sequenced isolates from the UK and Australia, with a low overall genomic distance (0.09%). Two isolates were notably different from the others and formed a clear separate clade: BTU-1 (isolated in 2012 from a host canid in Brazil) and ELAL-1 (isolated in 2019 from a host canid in TX, USA). Herein, we have described evidence for trans-boundary transmission of this virus in canid populations; ELAL-1 is very likely to have originated from a recombination event involving BTU-1. This discovery provides critical information for our collective understanding of the transmission of the virus and may play a future role in surveillance and control as the availability of whole viral genome sequencing increases. Despite robust import controls in the USA for live dogs and canine semen, there are presently no steps in place to test for CHV-1. Using a simple phylogeny assessment, we were able to create a visualization of the relationship between the CHV-1 isolates (Figure 2), which confirmed that isolates obtained from multiple host animals in the same outbreak in New York State (Figure 2b) were extremely closely related. Future sequenced isolates can be considered within this framework to determine likely phylogeny. Aside from this example of geography influencing clade organization, this effect appears to be much less pronounced for CHV-1 than for comparable viruses such as FHV-1, where geography appears to be a strong determinant of clade organization [21]. The high degree of similarity between the majority of CHV-1 genomes (Clade 1, Figure 2a) from host animals housed in a variety of geographic locations provides additional evidence for trans-boundary spread of this pathogen.

In the present study, two CHV-1 clades were detected by phylogenetic analysis. This is in agreement with previous analysis of 4 CHV-1 isolates; BTU-1 (Clade 2), 0194 (Clade 1), V777 (Clade 1) and V1154 (Clade 1) [20]. This previous work determined that the *UL50* gene of BTU-1 was 12.2% distant from the remaining isolates, hypothesized to be the result of a recombination event between CHV-1 and an unknown virus [20]. In the present study, the genome of ELAL-1 was found to be very similar to BTU-1 with one exception in the *V57* gene region. For comparison, the *V57* gene region of ELAL-1 was approximately 1.6% distant from both BTU-1 and 0194. BLAST searches (blast.ncbi.nlm.nih.gov) (Table S3) confirmed the closest identity of ELAL-1 *V57* to various other CHV-1 isolates, suggesting that positive selection is likely to be the underlying cause of this variation.

As most of the isolates were found to have homogenous genomes, it is unsurprising that variant detection (compared to 0194, the reference genome) yielded a modest number of results for each isolate. The exception was for ELAL-1, which contained 188 synonymous variants and 154 non-synonymous variants. For comparison, our set of CHV-1 isolates contained more variants than FHV-1 [21] but less than herpes simplex virus (HSV) [32,33]. As expected, most variants were found in larger regions of the CHV-1 genome (Table 3, Figure S2). It can be seen from the regression analysis in Figure S2 that a single gene (*UL50*, Deoxyuridine triphosphate) had a higher-than-expected total number of variants (53) relative to the gene size (918 bp). This gene had a relatively low number of variants in prior analyses of FHV-1 [21] and HSV-1 [34] and it is therefore unclear why this would be the case for CHV-1. Of possible clinical relevance is that one isolate (ELAL-1) contained variants in *UL23*, *UL30* and *UL42*, all regions targeted by antiviral medications [30]. Further investigation is necessary to determine if these variants have any impact on antiviral susceptibility. The host canid from which ELAL-1 was obtained had been treated by a veterinarian with multiple ocular medications for disease related to CHV-1 infection including topical ocular idoxuridine, without significant improvement of clinical signs. Following cessation of idoxuridine and initiation of topical ocular trifluridine [35], the ocular clinical signs improved. Antiviral resistance has been previously documented for HSV [36] but not for CHV-1.

The G-C content across the CHV-1 genomes was consistent at 31.6%. As has been previously noted [18,20], this is the lowest for known varicelloviruses. For comparison, the G-C content of FHV-1 is 45.8% and 72.6% for bovine herpes virus type 1 [20]. Sequencing coverage was excellent for CHV-1, and this is likely related to the consistently low G-C content across the genome for this virus [32,37]. It has been previously suggested that ungulate varicelloviruses, which have a higher G-C content, have a greater degree of intraspecies distance than those from non-ungulates (such as CHV-1). Our results fit within this framework, with a low overall distance of 0.09% for the 20 CHV-1 genomes analyzed.

We detected evidence of recombination between multiple isolates, a process which is thought to be common in herpesviruses [20,38,39]. In line with what has been previously reported, we did not detect any evidence of recombination between clades. By monitoring future occurrences of isolates from Clade 2, this should provide a useful method to monitor the spread of CHV-1 in the USA canid population. Recombination seems to be subjectively less prevalent in this sample of CHV-1 isolates compared to viruses such as FHV-1 [21] and HSV [32]. The reason for this is unknown.

All the CHV-1 isolates sequenced for the present study were obtained from the conjunctiva of animals with ocular disease (conjunctivitis and/or keratitis). Similar to other herpesviruses, CHV-1 establishes latency and is periodically excreted at various mucosal sites such as the conjunctiva, oral cavity and genitalia [40]. It is possible that the body sampling site influenced characteristics of the viral genomes which we obtained. This is considered unlikely given that many of the genomes from ocular isolates appeared to be very similar to those obtained from other tissues such as kidney, liver and lung.

## 5. Conclusions

Overall, our results agreed with previous reports of the genomics and phylogeny of CHV-1 [18,19,20]. We report further evidence of a novel form of CHV-1, recently isolated from a host canid located in the USA. Together these techniques can be used to elucidate the origins and transmission of this pathogen in domestic canid populations.

## Figures and Tables

**Table 1:** Details of isolates included in analysis. All isolates are canid alphaherpesvirus (CHV-1) apart from C-27, which is feline herpes virus 1 (FHV-1). M = Male, F = Female, FS = Female, spayed, MC = Male, castrated, ACS = American Cocker Spaniel, NA = not applicable/unknown.

Strain ID	Genbank Accession	Host Age/Sex/Breed	Host Location	Date Collected	Source Sample
ELAL-1	MW353125	7/FS/English Bulldog	San Antonio, TX, USA	6 March 2019	Eye
ELAL-2	MW353126	8/MC/Labrador Retriever	Oneonta, NY, USA	6 July 2003	Eye
ELAL-3	MW353127	0.33/F/Beagle	North Rose, NY, USA	19 November 2008	Eye
ELAL-4	MW353128	0.25/F/Beagle	North Rose, NY, USA	19 November 2008	Eye
ELAL-5	MW353129	7/FS/Min. Schnauzer	Endwell, NY, USA	26 August 2004	Eye
ELAL-6	MW353130	10/FS/Golden Retriever	Williston, VT, USA	17 September 2014	Eye
ELAL-7	MW353131	8/FS/Cross breed	Batavia, NY, USA	18 October 2007	Eye
ELAL-8	MW353132	8/MC/ACS	Ovid, NY, USA	25 February 2014	Eye
ELAL-9	MW353133	0.2/M/Beagle	North Rose, NY, USA	19 November 2008	Eye
ELAL-10	MW353134	0.2/M/Beagle	North Rose, NY, USA	19 November 2008	Eye
ELAL-11	MW353135	0.33/F/Beagle	North Rose, NY, USA	19 November 2008	Eye
ELAL-12	MW353136	12/MC/Cross breed	Wilton, CT, USA	16 October 2019	Eye
ELAL-13	MW353138	0.16/F/Beagle	Ithaca, NY, USA	16 April 2009	Eye
ELAL-14	MW353137	0.33/M/Beagle	North Rose, NY, USA	19 November 2008	Eye
ELAL-15	MW353139	9/FS/Golden Retriever	Binghamton, NY, USA	26 August 2008	Eye
V1154	KT819631	0.04/NA/Dalmatian	UK	2000	Kidney
V777	KT819632	0.04/NA/Min. Schnauzer	UK	1995	Lung
BTU-1	KX828242	NA/NA/NA	Brazil	2012	Kidney
15-4016-NSW	KY057364	0.08/F/Labrador Retriever	NSW, Australia	2016	Liver
0-194	NC_030117	NA/NA/NA	NA	1985	NA
C-27	NC_013590	NA/NA/NA	NA	Pre 1980	NA

**Table 2:** Sequencing details of sequenced CHV-1 isolates.

Strain ID	Number Reads	Mapped Reads	Mean Coverage	Mean Mapped Read Length (bp)
ELAL-1	2,865,972	155,016	290.2	226.9
ELAL-2	2,963,230	390,228	706.8	228.6
ELAL-3	1,292,262	231,149	413.2	225.3
ELAL-4	1,848,000	376,806	663.9	223.1
ELAL-5	1,430,708	279,714	476.8	214.7
ELAL-6	3,053,006	847,371	1509.6	225.4
ELAL-7	3,221,498	219,157	372.8	214.5
ELAL-8	1,889,752	298,155	505.4	213.2
ELAL-9	3,415,468	416,370	706	213.7
ELAL-10	3,533,658	648,209	1409.8	201.2
ELAL-11	2,356,364	456,166	765.2	211.7
ELAL-12	3,126,278	639,677	1024.4	201.1
ELAL-13	2,496,354	326,583	536.8	206.8
ELAL-14	2,955,844	856,992	1404	207.5
ELAL-15	2,985,658	527,658	870.8	205.7

**Table 3:** Summary of variants detected, organized by gene involved. F = Forward, R = Reverse.

Gene	Gene Product	Number Unique Synonymous Variants	Number Unique non-Synonymous Variants	Length of Gene (bp)
*Circ*	Myristylated tegument protein CIRC	4	2	651
*RL2*	Ubiquitin E3 ligase ICP0	1	4	1005
*RS1*	Transcriptional regulator ICP4	22	16	4155(F)/4155(R)
*UL1*	Envelope glycoprotein L	0	0	468
*UL10*	Envelope glycoprotein M	1	2	1254
*UL11*	Myristylated tegument protein	1	0	213
*UL12*	Deoxyribonuclease	2	0	1638
*UL13*	Tegument serine/threonine protein kinase	2	5	1770
*UL14*	Tegument protein UL14	1	4	912
*UL15*	DNA packaging terminase subunit 1	1	2	5442
*UL16*	Tegument protein UL16	4	2	1092
*UL17*	DNA packaging tegument protein UL17	0	4	2067
*UL18*	Capsid triplex subunit 2	3	0	948
*UL19*	Major capsid protein	7	3	4122
*UL2*	Uracil-DNA glycosylase	0	0	837
*UL20*	Envelope protein UL20	2	0	693
*UL21*	Tegument protein UL21	2	1	1572
*UL22*	Envelope glycoprotein H	1	1	2394
*UL23*	Thymidine kinase	0	2	987
*UL24*	Nuclear protein UL24	1	0	783
*UL25*	DNA packaging tegument protein UL25	2	1	1758
*UL26*	Capsid maturation protease	4	2	1731
*UL26.5*	Capsid scaffold protein	3	1	846
*UL27*	Envelope glycoprotein B	1	1	2640
*UL28*	DNA packaging terminase subunit 2	3	2	2271
*UL29*	Single-stranded DNA-binding protein	5	4	3594
*UL3*	Nuclear protein UL3	0	1	597
*UL30*	DNA polymerase catalytic subunit	2	4	3540
*UL31*	Nuclear egress lamina protein	1	1	1005
*UL32*	DNA packaging protein UL32	1	2	1680
*UL33*	DNA packaging protein UL33	0	1	381
*UL34*	Nuclear egress membrane protein	1	1	795
*UL35*	Small capsid protein	0	0	321
*UL36*	Large tegument protein	15	42	9531
*UL37*	Tegument protein UL37	3	4	3039
*UL38*	Capsid triplex subunit 1	1	0	1374
*UL4*	Nuclear protein UL4	3	1	663
*UL41*	Tegument host shutoff protein	0	4	1461
*UL42*	DNA polymerase processivity subunit	1	2	1059
*UL43*	Envelope protein UL43	3	2	1203
*UL44*	Envelope glycoprotein C	1	1	1380
*UL45*	Membrane protein UL45	1	1	528
*UL46*	Tegument protein VP11/12	0	1	1986
*UL47*	Tegument protein VP13/14	7	8	2439
*UL48*	Transactivating tegument protein VP16	3	1	1269
*UL49*	Tegument protein VP22	7	1	795
*UL49A*	Envelope glycoprotein N	3	1	261
*UL5*	Helicase-primase helicase subunit	1	3	2661
*UL50*	Deoxyuridine triphosphate	20	33	918
*UL51*	Tegument protein UL51	14	6	672
*UL52*	Helicase-primase primase subunit	17	14	3063
*UL53*	Envelope glycoprotein K	8	4	1014
*UL54*	Multifunctional expression regulator	4	0	1272
*UL55*	Nuclear protein UL55	3	1	564
*UL56*	Membrane protein UL56	0	5	522
*UL6*	Capsid portal protein	8	1	2073
*UL7*	Tegument protein UL7	2	2	879
*UL8*	Helicase-primase subunit	6	3	2271
*UL9*	DNA replication origin-binding helicase	3	3	2535
*US1*	Regulatory protein ICP22	5	1	942(F)/942(R)
*US10*	Virion protein US10	1	5	588(F)/588(R)
*US2*	Virion protein US2	3	2	1176
*US3*	Serine/threonine protein kinase US3	0	0	1203
*US4*	Envelope glycoprotein G	1	1	1248
*US6*	Envelope glycoprotein D	3	1	1038
*US7*	Envelope glycoprotein I	3	1	1038
*US8*	Envelope glycoprotein E	2	4	1569
*US8A*	Membrane protein US8A	0	0	237
*US9*	Membrane protein US9	0	2	360
*V32*	Protein V32	0	2	417
*V57*	Protein V57	1	1	729
*V67*	Virion protein V67	6	0	552(F)/552(R)

## Supplementary Materials

The following are available online at https://www.mdpi.com/1999-4915/12/12/1421/s1, Figure S1: Coverage (in blue) of a representative isolate (ELAL-7) along the length of the genome. A schematic of the genome is shown immediately below, with regions of low coverage (less than 100x) identified with red bars. Figure S2: Total number of variants per gene detected in CHV-1 isolates sequenced for this study plotted against the length (bp) of each gene. The solid blue line represents the linear trendline with 99% confidence intervals shown with dotted black lines. The red data point indicates the single gene where the number of detected variants was above the expected number of variants (UL50/53 variants/918 bp). Table S1: Unique non-synonymous variants organized according to gene region affected. Table S2: Results of manual bootscan in RDP4 for each CHV-1 isolate analyzed. Table S3: Results of BLAST for ELAL-1 V57 region against nt database using BLASTN (blast.ncbi.nlm.nih.gov).
