# Supplementary figures and images for: Phylogenomic Analysis of Global Isolates of Canid Alphaherpesvirus 1
Source: Viruses. 2020 Dec 10;12(12):1421. doi: 10.3390/v12121421 (PMC7764265; doi:10.3390/v12121421)

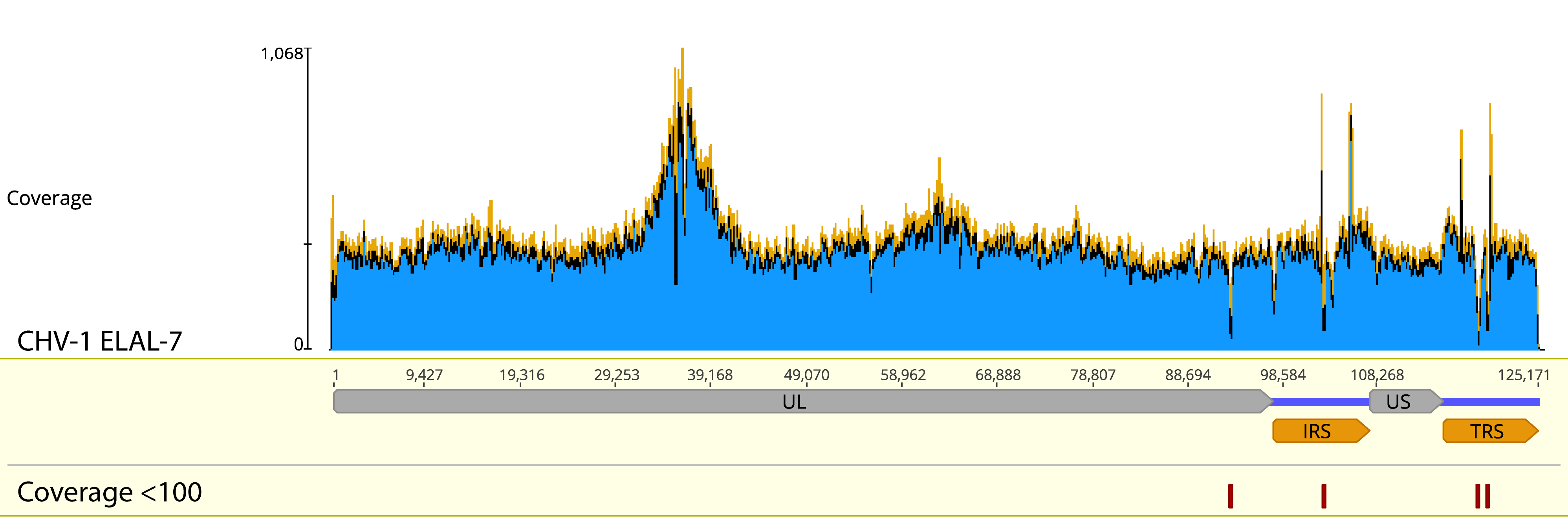

Supplement: Supplementary file 1 [file viruses-12-01421-s001.zip › Figure S1.tif]

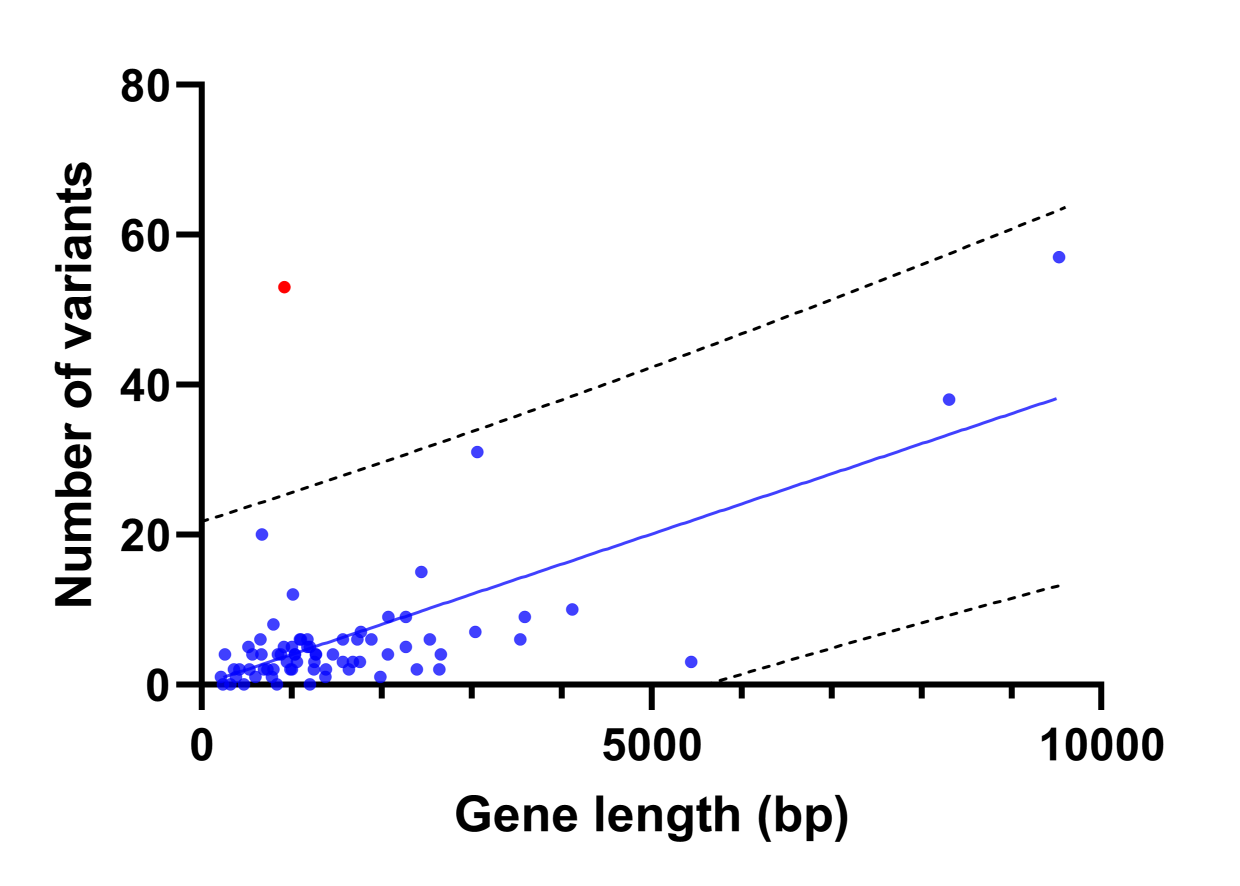

Supplement: Supplementary file 1 [file viruses-12-01421-s001.zip › Figure S2 12.11.20.tif]
